# Suppression of Continuous Wave Interference in Loran-C Signal Based on Sparse Optimization Using Tunable Q-Factor Wavelet Transform and Discrete Cosine Transform

**DOI:** 10.3390/s21217153

**Published:** 2021-10-28

**Authors:** Wenwen Ma, Jiuxiang Gao, Yanning Yuan, Zhensheng Shi, Xiaoli Xi

**Affiliations:** 1 School of Information and Communications Engineering, Xi’an Jiaotong University, Xi’an 710049, China; m2w1001@stu.xjtu.edu.cn; 2 School of Automation and Information Engineering, Xi’an University of Technology, Xi’an 710048, China; 1190311014@stu.xaut.edu.cn (J.G.); yyn1982@126.com (Y.Y.); xixiaoli@xaut.edu.cn (X.X.)

**Keywords:** Loran-C, continuous wave interference, wavelet transform, discrete cosine transform, morphological component analysis

## Abstract

Loran-C is the most essential backup and supplementary system for the global navigation satellite system (GNSS). Continuous wave interference (CWI) is one of the main interferences in the Loran-C system, which will cause errors in the measurement of the time of arrival, thereby affecting positioning performance. The traditional adaptive notch filter method needs to know the frequency of CWI when removing it, and the number is limited. This paper presents a method based on sparseness to suppress the CWI in the Loran-C signal. According to the different morphological characteristics of the Loran-C signal and the CWI, we construct dictionaries suitable for the two components, respectively. We use the tunable Q-factor wavelet transform and the discrete cosine transform to make the two components obtain a good sparse representation in their respective dictionaries. Then, the two components are separated using the morphological component analysis theory. We illustrate this method using both synthetic data and actual data. A huge advantage of the proposed method is that there is no need to know the frequencies of the CWI for it can better cope with frequency changes of the CWI in the actual environments. Compared with the adaptive notch filter method, the results of the proposed method show that our approach is more effective and convenient.

## 1. Introduction

Loran-C is a land-based hyperbolic radio positioning, navigation, and timing (PNT) international standardized system [1,2]. It has the characteristics of long propagation distance, high transmitting power, and good stability [3]. Recently, with the potential vulnerability of the global navigation satellite system (GNSS), such as security threat, renewed interest in Loran-C applications and development has appeared [4,5,6,7]. It is ideally suited to serve as a reliable backup and secure complement for the GNSS as its signal generation and transmission are quite different and completely independent from the GNSS, which can provide redundant capability in the event that the GNSS signals are disrupted [8,9,10]. Therefore, it not only reduces critical dependencies on the GNSS but also ensures the availability of uncorrupted and non-degraded PNT signals for military and civilian users. Today, numerous countries and regions have deployed Loran systems to provide a resilient and reliable alternative positioning and timing system to meet military and civil needs, such as the United States, China, Russia, South Korea, Europe, etc. [11,12,13,14]. In a nutshell, the Loran-C system will continue to occupy a key position in the future. Moreover, the Loran-C signal processing technology also needs to be further improved to meet the demands of modern PNT services [15].

The Loran-C system works in the low-frequency band and is susceptible to interference from high-power communication stations. This interference is called continuous wave interference (CWI). The Loran-C system locates based on the difference of the time of arrival (TOA) of pulses emitted by different transmitters, including tracking a specific zero-crossing point [16]. However, the CWI can contaminate the Loran-C signal and affect TOA measurement [17,18]. It will cause the TOA measurement to be inaccurate and eventually lead to incorrect positioning. To improve the performance of the Loran-C receiver, it is necessary to remove the interference and noise of the received signal as much as possible. CWI can be divided into three main types: synchronous, near-synchronous, and asynchronous interference [19]. Asynchronous CWI can be suppressed by using a bandpass filter in the Loran-C receiver. However, because the frequency of synchronous and near-synchronous CWI is within the bandwidth of the Loran-C signal and the bandwidth of CWI is very narrow, it is easy to damage the effective signal when suppressing these two types of CWI. At present, the traditional solution for the CWI is to use adaptive notch filters with a limited number of fixed frequencies in the Lora−C receiver [20]. The notch filter technique has a very high requirement for frequency estimation [21]. In an increasingly complex electromagnetic environment, the frequency of the CWI varies with the environment. However, the number of notch filters is fixed and cannot be changed with the environment. In addition, if the frequency estimate deviates, the notch filter will lose its function. Therefore, it is essential to develop a method of CWI suppression that does not require known CWI frequencies.

In [22], a sparse representation method for separating the image into texture and piecewise parts by the morphological component analysis (MCA) theory has been proposed. They used two appropriate dictionaries to represent different parts and make them sparsely represented on the component they apply while not sparsely represented on the other content. This method provides a new idea for separating different components of signals according to their different characteristics. Recognizing the superiority of the MCA method, we considered the suppression of CWI based on signal sparse representation. Selecting suitable dictionaries in the MCA theory can greatly improve the effectiveness of the separation of signal and interference. The tunable Q-factor wavelet transform (TQWT) is presented in [23]. It can match the oscillating characteristics of the signal by using the different Q-factor, thereby enhancing the sparsity when using it for signal sparse representation [24]. TQWT has been used in many areas [25,26,27]. It also has a good application prospect in Loran-C signals. A discrete cosine transform (DCT) is often used in the area of digital processing [28]. It is also one of the dictionaries commonly used for sparse representation. According to the characteristics of CWI, it is suitable to sparsely represent CWI using DCT.

This paper proposes a novel method to suppress the CWI of the Loran-C signal. The MCA theory is used to separate the two components. The Loran-C signal has high oscillation characteristics in the time domain and thus can be used with a dictionary formed by the tunable Q-factor wavelet transform. DCT has a good sparse representation of continuous waves of a single frequency, so the sparse representation dictionary constituted by the DCT can represent the CWI. Consequently, the CWI can be suppressed by iterative optimization. Thus, we can suppress it without prior knowledge of the CWI frequencies. Moreover, our method is more efficient than traditional methods and can even suppress white noise. Therefore, we have developed a simple and effective method to remove the CWI in Loran-C signal automatically.

## 2. Materials and Methods

**Figure 1:**
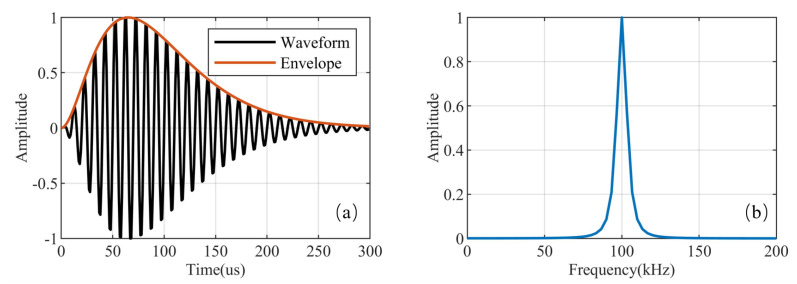
The characteristics of a single pulse of Loran-C of a positive phase code: (**a**) Loran-C pulse waveform; (**b**) Loran-C pulse spectrum.

The Loran C signals are transmitted in the form of a series of pulse groups [29]. A single ideal Loran-C pulse is defined by   

(1) $$\begin{array}{c} i\left(t\right)=\left\{\begin{array}{c} \;0\;\;\;\;\;\;\;\;\;\;\;\;\;\;\;\;\;\;\;\;\;\;\;t<\tau_{d}\\ A{\left(t\;-\;\tau_{d}\right)}^{2}exp\;\left[\;-\frac{2\left(t\;-\;\tau_{d}\right)}{65}\right]\;sin\;\left(0.2\pi t\;+\;P_{C}\right)\;\tau_{d}\leq t\leq 65\;+\;\tau_{d},\\ \;\;Undefined\;\;\;\;\;\;\;\;\;\;\;\;\;\;\;t>65\;+\;\tau_{d} \end{array}\right. \end{array}$$

where *A* is the normalization constant, $\tau_{d}$ is the envelope-to-cycle difference (ECD), and *Pc* is the phase code parameter, which takes the value 0 for a positive phase code or π for a negative phase code. In addition, the carrier frequency of the Loran-C signal is 100 kHz, and its envelope is defined by$\;A{\left(t-\tau_{d}\right)}^{2}exp\left[-\frac{2\left(t-\tau_{d}\right)}{65}\right]$. The waveform and frequency spectrum of a single pulse of a positive phase code is shown in Figure 1. As can be seen from the Figure 1, the duration of a single Loran-C pulse is 260 μs, and 99% of its energy is concentrated in the 90–110 kHz band [30,31].

### 2.1. Signal and Interference Separation Model

**Figure 2:**
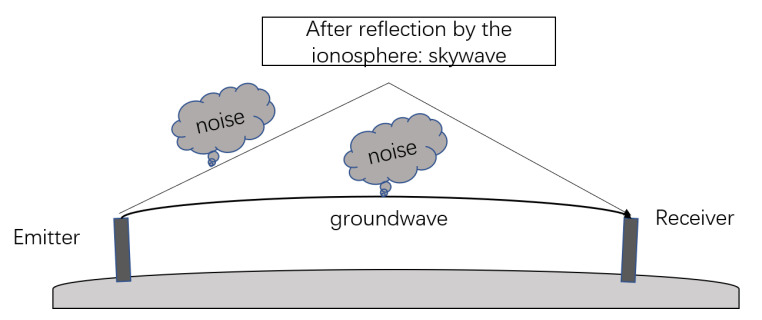
The propagation of skywave and groundwave.

The Loran-C signal received by the receiver can be expressed by

(2) $$s=s_{g}+s_{s}+s_{c}+n,$$

where $s_{g}$ stands for groundwave, $s_{s}$ for skywaves, and $s_{c}$ for the CWI and *n* for noise. Figure 2 shows the propagation of the skywave and groundwave. Groundwaves propagate parallel to the earth’s surface, while skywaves travel upward and reflect through the ionosphere. The skywave time delay is generally between 35 μs and 200 μs, so the groundwave in the latter part of the Loran-C received signal will overlap with the skywaves. Moreover, skywaves and groundwaves differ only in amplitude and time of arrival. For this reason, the skywaves and the groundwaves can be regarded as one component. Thus, the received Loran-C signal can also be represented by

(3) $$s=s_{l}+s_{c}+n,$$

where $s_{l}$ stands for groundwave and skywaves, $s_{c}$ for CWI, and *n* for noise. Morphological component analysis (MCA) can successfully separate the two components by choosing the appropriate sparse representation dictionaries that represent the different components of the signal, respectively. The selection of sparse representation dictionaries is closely related to the morphological characteristics of signal components. Since the morphological characteristics of $s_{l}$ and $s_{c}$ are different, the conditions for suppressing CWI in the signal received by the Loran-C receiver are consistent with the MCA method. The separation can be achieved by solving the constraint minimization problem:

(4) $$\begin{array}{c} \left\{x_{l}^{opt},x_{c}^{opt}\right\}=\underset{\left\{x_{l},x_{c}\right\}}{\arg\min}{∥x_{l}∥}_{1}+{∥x_{c}∥}_{1}s.t.{∥s-\Phi_{l}x_{l}-\Phi_{c}x_{c}∥}_{2}^{2}\leq \varepsilon , \end{array}$$

where $\Phi_{l}$ and $\Phi_{c}$ are sparse representation dictionaries that can only sparsely represent skywave and groundwave component $s_{l}$ and CWI component $s_{c}$, respectively. $x_{l}$ and $x_{c}$ are the sparse coefficients representing $s_{l}$ and $s_{c}$. ε is the error threshold of the signal reconstruction. The successful separation of the two components of the signal depends on the choice of the dictionaries. The dictionaries should be able to sparsely represent the corresponding signal component and not sparsely represent the other component. For an appropriate Lagrange multiplier, the problem can become unconstrained:

(5) $$\begin{array}{c} \left\{x_{l}^{opt},x_{c}^{opt}\right\}=\underset{\left\{x_{l},x_{c}\right\}}{\arg\min}\frac{1}{2}{∥s-\Phi_{l}x_{l}-\Phi_{c}x_{c}∥}_{2}^{2}+\lambda ({∥x_{l}∥}_{1}+{∥x_{c}∥}_{1}). \end{array}$$

The above optimization objective problem can be solved by the algorithm based on the block-coordinate-relaxation (BCR) method [32]. In Algorithm 1, $\Phi_{c}^{*}$ and $\Phi_{l}^{*}$ are the forward transformations of $\Phi_{c}$ and $\Phi_{l}$. We can obtain the Loran-C signal component with suppressed CWI through the algorithm. The two specific dictionary selections and details will be described in the following sections.

The two components have different amounts of energy, so we use different initial thresholds. The threshold coefficient λ takes a number close to but less than 1. We obtained the final sparse representation coefficients of the Loran-C signal and the CWI through iteration computation and the threshold mechanism. Then using the corresponding dictionary, we can obtain the two separated components.
**Algorithm 1** Block-Coordinate-Relaxation.**Input:** the number of iterations *N*, the received signal *s*, the threshold coefficient λ; **Output:** Loran-C signal component $s_{l}$, continuous wave interference component $s_{c}$;
 Initialize: $x_{l}^{0}\leftarrow 0$, threshold $T_{c}$, threshold $T_{l}$;
 **for** 
k=0 
*N* **do**
  $x_{c}^{k+1}\leftarrow T_{c}\left(\Phi_{c}^{*}\left(s-\Phi_{l}x_{l}^{k}\right)\right)$
  $x_{l}^{k+1}\leftarrow T_{l}\left(\Phi_{l}^{*}\left(s-\Phi_{c}x_{c}^{k+1}\right)\right)$
  $T_{c}\leftarrow \lambda T_{c}$
  $T_{l}\leftarrow \lambda T_{l}$
 **end for**
 $s_{l}\leftarrow \Phi_{l}x_{l}^{N}$
 $s_{c}\leftarrow \Phi_{c}x_{c}^{N}$
 **return** $s_{l}$, $s_{c}$;


### 2.2. Sparse Representation Dictionary Construction Strategy

In the MCA model, constructing suitable dictionaries for different components is the key to successfully separating the two constituents of the signal. We chose the tunable Q-factor wavelet transform (TQWT) with Q=10 and the discrete cosine transform (DCT) as the sparse representation dictionaries of the Loran-C signal and the CWI, respectively. Next, we will describe the two dictionaries.

#### 2.2.1. Tunable Q-Factor Wavelet Transform

TQWT can adjust the value of the Q-factor to adapt to signals with different oscillation behaviors, thereby improving the sparsity of signal representation. The Q-factor affects the duration of wavelet oscillation. The higher the *Q* coefficient, the longer the wavelet oscillation lasts. The Q-factor is defined as the ratio of the pulse center frequency $w_{c}$ to the bandwidth BW:

(6) $$Q=\frac{w_{c}}{BW},$$

the bandwidth here refers to half of the non-zero interval in the frequency response.

We can improve the sparsity of the Loran-C signal representation by setting an appropriate Q-factor to match the oscillating behavior of the Loran-C signal. Since the center frequency of the Loran-C signal is 100 kHz, the bandwidth is 10 kHz. Therefore, Q=10 is suitable for the morphological characteristics of skywaves and groundwaves.

**Figure 3:**
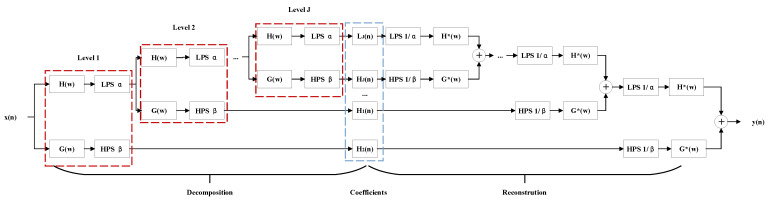
The analysis and synthesis filter banks for TQWT.

As shown in Figure 3, TQWT is implemented by iterating the two-channel filter bank over its low-pass channel. To achieve perfect signal reconstruction, the input signal x(n) of the filter bank needs to be equal to the reconstructed output signal y(n). The frequency responses of the low-pass filter H(w) and the high-pass filter G(w) are generally defined by:

(7) $$H\left(w\right)=\left\{\begin{array}{l} 1,\;\;\;\;\;\;\;\;\;\;\;\;\;\;\;\;\;\;\;\;\;\;\left|w\right|\leq \left(1-\beta \right)\pi \\ \theta \left(\frac{w+(\beta -1)\pi }{\alpha +\beta -1}\right),\;\;\;\;\;\left(1-\beta \right)\pi <\left|w\right|<\alpha \pi \\ 0,\;\;\;\;\;\;\;\;\;\;\;\;\;\;\;\;\;\;\;\;\;\;\;\alpha \pi \leq \left|w\right|\leq \pi , \end{array}\right.$$

and

(8) $$G\left(w\right)=\left\{\begin{array}{c} 0,\;\;\;\;\;\;\;\;\;\;\;\;\;\;\;\;\;\;\;\;\;\;\;\;\;\;\left|w\right|\leq \left(1-\beta \right)\pi \\ \theta \left(\frac{\alpha \pi -\omega }{\alpha +\beta -1}\right),\;\;\;\;\;\;\;\;\;\;\;\;(1-\beta )\pi <\left|w\right|<\alpha \pi \\ 1,\;\;\;\;\;\;\;\;\;\;\;\;\;\;\;\;\;\;\;\;\;\;\;\;\;\;\alpha \pi \leq \left|w\right|\leq \pi , \end{array}\right.$$

where

(9) $$\theta \left(w\right)=0.5\left(1+cosw\right)\sqrt{2-cosw},\;\;\;\;\;\;\;\left|w\right|\leq \pi$$

is the Daubechies frequency response with two vanishing moments as in [33], α is the low-pass scaling parameter, and β is the high-pass scaling parameter. The frequency response satisfies the conditions of a perfect reconstruction ${\left|H(w)\right|}^{2}+{\left|G(w)\right|}^{2}=1$. The scaling parameters need to satisfy 0<β≤1,0<α≤1 to make sure the wavelet transform will not be overly redundant. In order to perfect reconstruction, its required that α+β>1. Low-pass scaling preserves the low-frequency content of the signal in frequency-domain scaling. Specifically, when 0<α≤1, low-pass scaling is defined as:

(10) $$Y\left(w\right)=X\left(\alpha w\right),\;\;\;\;\left|w\right|\leq \pi ,$$

in contrast, high-pass scaling preserves the high-frequency content of the signal in frequency-domain scaling. Specifically, when 0<β≤1, high-pass scaling is defined as:

(11) $$Y\left(w\right)=X\left(\beta w+\frac{w}{\left|w\right|}\left(1-\beta \right)\pi \right),\;\;\;\;0<\left|w\right|<\pi ,$$

we can express the main parameters α and β through the Q-factor and redundancy in the filter banks:

(12) $$\beta =\frac{2}{Q+1},\alpha =1-\frac{\beta }{r},$$

where *Q* is a positive integer, and *r* is the total oversamping rate, generally an integer greater than or equal to 3. Therefore, the dominating parameter of the tunable Q-factor wavelet transform is the Q-factor.

**Figure 4:**
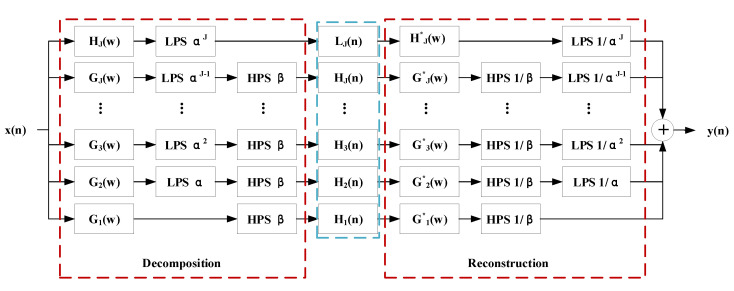
The equivalent filter banks for TQWT.

We have the equivalent system shown in Figure 4, and the equivalent frequency responses are expressed as:

(13) $$H_{j}\left(w\right)=\left\{\begin{array}{l} \prod_{m=0}^{j-1}H(w/\alpha^{m}),\;\;\;\left|w\right|\leq \alpha^{j}\pi \\ \;\;0,\;\;\;\;\;\;\;\;\alpha^{j}\pi <\left|w\right|\leq \pi \end{array}\right.,$$

and

(14) $$\begin{array}{c} G_{j}\left(w\right)=\left\{\begin{array}{c} \;G\left(w/\alpha^{j-1}\right)\;\prod_{m=0}^{j-2}G\left(w/\alpha^{m}\right),\;\left(1\;-\;\beta \right)\alpha^{j-1}\pi \;\leq \;\;\left|w\right|\;\;\leq \;\;\alpha^{j-1}\pi \\ \;\;0,\;\;\;\;\;\;\;\;\;\;\;\mathrm{for}\;\mathrm{other}\;w\in \;\left[-\pi ,\pi \right] \end{array}\right.. \end{array}$$

#### 2.2.2. Discrete Cosine Transform

DCT is one of the commonly used transformations in the field of digital signal processing. It is also one of the popular sparse representation dictionaries. The morphological characteristics of CWI are similar to cosine waves, and the frequency bandwidth is very narrow. Thus, we can improve the sparsity of the CWI representation by using the discrete cosine transform. The DCT is defined as:

(15) $$\begin{array}{c} X_{C}\left[k\right]=w\left(k\right)\sum_{n=1}^{N}x\left[n\right]\cos\frac{\pi (2n-1)(k-1)}{2N},k=1,2,\cdots ,N, \end{array}$$

where x[n] represents the signal to be transformed, *N* represents the signal length, $X_{C}\left[k\right]$ represents the discrete cosine transform coefficient, and w(k) is:

(16) $$w\left(k\right)=\left\{\begin{array}{c} \frac{1}{\sqrt{N}},k=1\\ \sqrt{\frac{2}{N}},k\neq 1 \end{array}\right\}.$$

The Inverse Discrete Cosine Transform (IDCT) is defined as:

(17) $$\begin{array}{c} x\left[n\right]=\sum_{k=1}^{N}w(k)X_{C}\left[k\right]\cos\frac{\pi (2n-1)(k-1)}{2N},n=1,2,\cdots ,N. \end{array}$$

According to the different morphological characteristics of the Loran-C signal and CWI, we constructed dictionaries suitable for them, respectively. Then, we use the BCR algorithm mentioned above to separate the two components. In this way, we achieved CWI suppression in the Loran-C signal.

## 3. Results and Discussion

In this section, we apply the proposed method to synthetic data and actual data. In addition, we analyzed the performance and advantages of the proposed method and compared it with the adaptive notch filter method.

### 3.1. Synthetic Data Example

**Figure 5:**
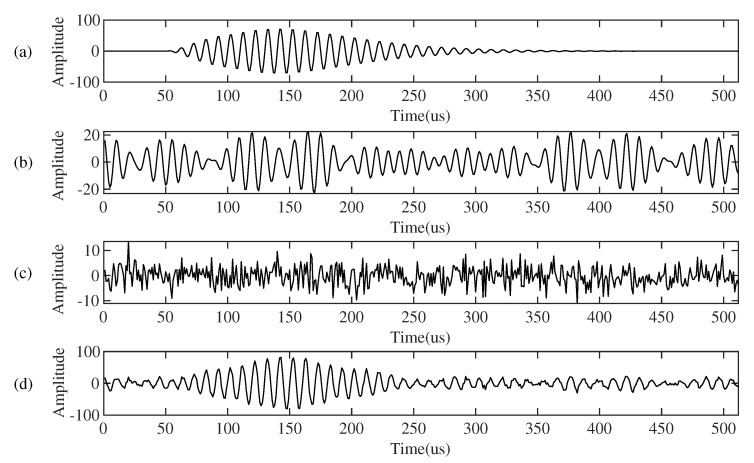
The simulation signal. (**a**) Simulated Loran-C signal. (**b**) Simulated continuous wave interference. (**c**) Gaussian white noise. (**d**) Composite signal.

In order to prove that the proposed method can effectively suppress continuous wave interference (CWI), we first use synthetic data to verify. Figure 5 shows the simulation data we used. Figure 5a is the simulated Loran-C signal, which is composed of the groundwave and the skywave delayed by 50 us relative to the groundwave. The amplitude ratio of the skywave and the groundwave is 1:2. Figure 5b shows the added continuous wave interference with frequencies of 85 kHz, 90 kHz, 93 KHz, and 109 kHz. The signal-to-interference ratio is 7.5 dB. Specifically, the CWI of each frequency is described in Equation (18).

(18) $$\left\{\begin{array}{l} i_{1}\left(t\right)=50\cos(2\pi *85t+\pi /6)\\ i_{2}\left(t\right)=70\sin\left(2\pi *90t+\pi /2\right)\\ i_{3}\left(t\right)=65\sin\left(2\pi *93t+\pi /3\right)\\ i_{4}\left(t\right)=75\sin\left(2\pi *109t+\pi /5\right). \end{array}\right.$$

Figure 5c is the added Gaussian white noise, and the signal-to-noise ratio is 15 dB, while Figure 5d is the synthesized signal we used, which is composed of the simulated Loran-C signal, the simulated CWI, and noise.

In the simulation data, TQWT with Q=10 and DCT are selected as the sparse representation dictionary of the Loran-C signal and the CWI, respectively. Table 1 illustrates the sparsity estimation of different components using different dictionaries. We use the zero norm of the sparse representation coefficients to represent the sparsity, and the smaller the value, the higher the sparsity. The sparsity of TQWT with Q=10 for the Loran-C signal is 0.0214, while that for the CWI is 0.0764. It is shown that the Loran-C signal represented by the TQWT dictionary has better sparsity than representing the CWI. Similarly, using DCT to indicate sparsity for the CWI is 0.0156, while that for the Loran-C signal is 0.0195. The CWI represented by the DCT dictionary has better sparsity than representing the Loran-C signal. According to the sparsity results, it can be concluded that TQWT with Q=10 is suitable for use as the sparse representation dictionary of the Loran-C signal, while DCT is suitable for the CWI. Through the sparsity estimation, we verified the correctness of the sparse representation dictionary construction strategy.

**Figure 6:**
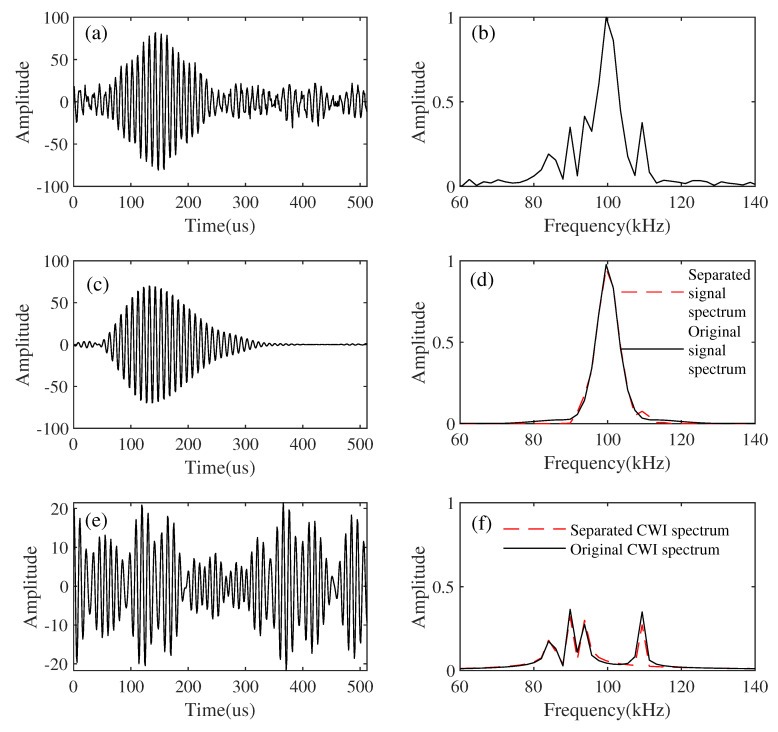
Results of separation by the proposed method (white noise: 15 dB). (**a**) Simulated synthetic signal. (**b**) Spectrum of simulated synthetic signal. (**c**) Separated Loran-C signals. (**d**) The spectrum of separated Loran-C signal. (**e**) Separated CWI. (**f**) The spectrum of separated CWI.

The proposed method is applied to the synthetic data example using the constructed dictionaries. Figure 6c,e show the separated Loran-C signal and the CWI, respectively. Comparing Figure 6a, we can see that the CWI is effectively suppressed, and at the same time, the effective signal is well preserved. The proposed method can also suppress white Gaussian noise. A spectrum comparison can also verify this well. Figure 6b is the spectrum of the simulated synthesized signal. We can observe the added CWI frequencies through the spectrum. As is shown in Figure 6d, the red line is the spectrum of the separated Loran-C signal, and the black one is the spectrum of the original Loran-C signal form, with almost no damage to the effective signal. As Figure 6e shows, the separated CWI spectrum is represented by the red line and that of the original CWI spectrum used a black line, from which we can see that the CWI of 85 kHz, 90 KHz, 93 kHz, and 109 kHz have been effectively suppressed.

**Figure 7:**
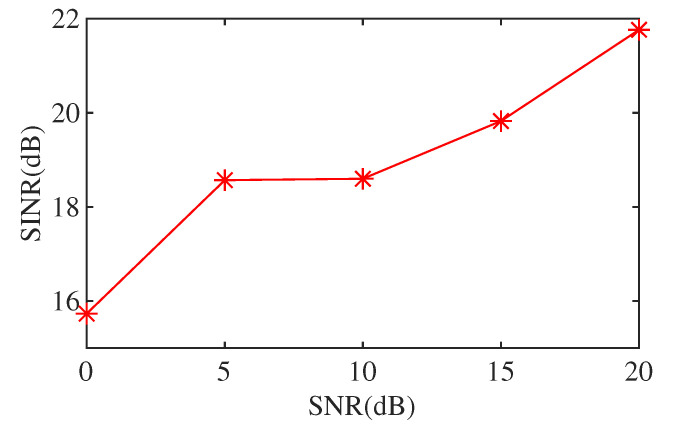
The change of SINR of results after adding white noise with different SNR.

**Figure 8:**
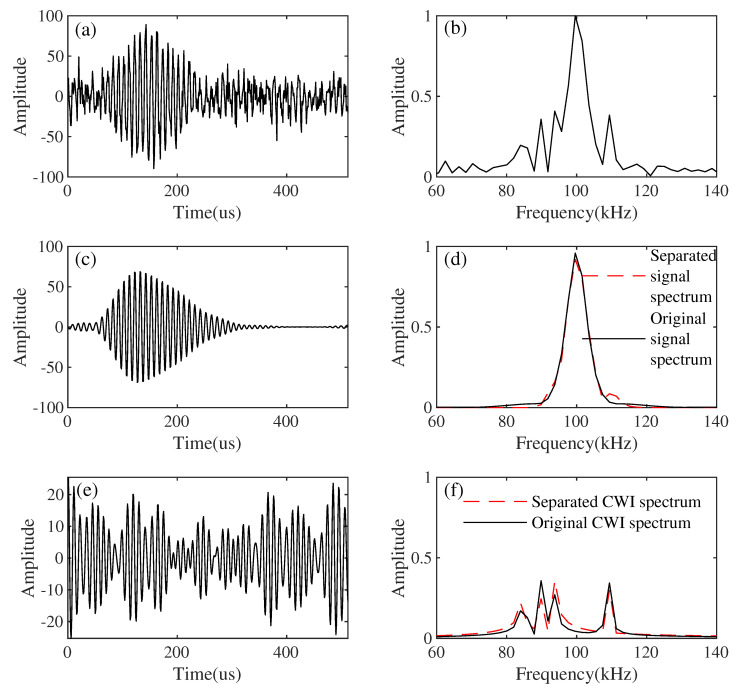
Results of separation by the proposed method (white noise: 5 dB). (**a**) Simulated synthetic signal. (**b**) Spectrum of simulated synthetic signal. (**c**) Separated Loran-C signals. (**d**) The spectrum of the separated Loran-C signal. (**e**) Separated CWI. (**f**) The spectrum of the separated CWI.

In order to illustrate the influence of Gaussian white noise on the performance of the proposed method, we added white noise with different signal-to-noise ratios for comparison. Figure 7 shows the signal-to-interference plus noise ratio (SINR) of the results obtained by the proposed method when adding white noise with different SNR to the input. The added interference is the same. When SNR is low, the performance of the proposed algorithm may degrade. When the SNR is greater than 5 dB, the proposed method will not cause the loss of useful signals. We can verify this in Figure 8. We add Gaussian white noise with SNR of 5 dB, and the result is shown in Figure 8. Figure 8a,b are the simulated synthesized signal and its spectrum. Figure 8c,d show the separated Loran-C signal and its spectrum, respectively. It can be seen from Figure 8d that the effective signal is not damaged. Figure 8e,f show the separated CWI and its spectrum, respectively. It can be seen that the CWI of 85 kHz, 90 KHz, 93 kHz, and 109 kHz have been effectively suppressed.

The Loran-C system generally locates by tracking the third zero-crossing point of the pulse. As shown in Table 2, we compared the position of the third zero-crossing point after adding white noise with different SNRs. The added interference is the same as in Figure 5b. It can be observed from the table that the third zero-crossing position of the processed signal is closer to the original signal than that of the signal with noise and interference added. In addition, noise and interference affect the detection of the third zero crossing of the Loran-C signal. When the useful signal is drowned out by interference and noise, it is difficult to tell which zero crossing is of the Loran-C signal. Therefore, the method proposed in this paper can improve the positioning accuracy.

**Figure 9:**
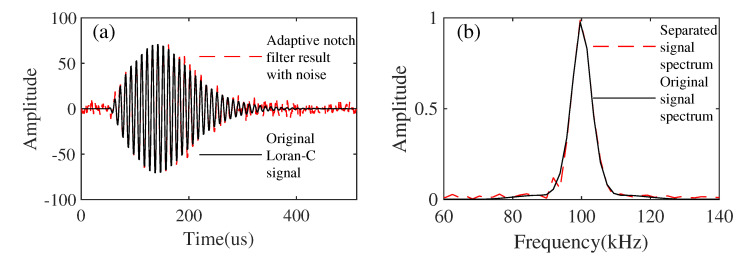
The results of CWI suppression using an adaptive notch filter with noise added. (**a**) The results of the adaptive notch filter with noise added. (**b**) Comparison of the separated signal spectrum and the original signal spectrum.

**Figure 10:**
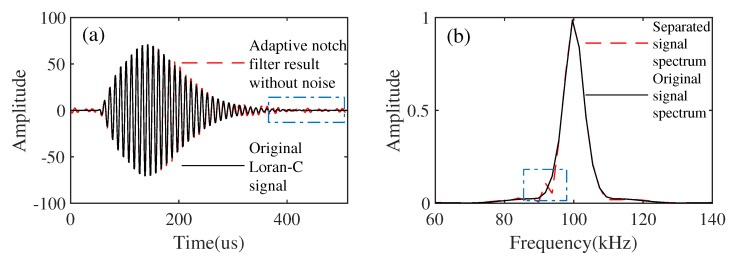
The results of CWI suppression using an adaptive notch filter without noise added. (**a**) The results of adaptive notch filter without noise. (**b**) Comparison of the separated signal spectrum and the original signal spectrum.

The comparison with the adaptive notch filter method can also illustrate the effectiveness and advantages of the proposed method. As is shown in Figure 9a, this is the result of removing the CWI from the simulated synthesized data using the adaptive notch filter approach. Figure 9b is the comparison of the separated signal spectrum and the original signal spectrum. The proposed method can also remove Gaussian white noise successfully, but the adaptive notch filter cannot. In this case, it is difficult to compare the two methods. Therefore, we used the adaptive notch filter method to suppress the CWI in the simulated synthetic data without white noise added in order to make a valid comparison. Figure 10a shows the result of suppressing the CWI from the synthesized data without noise added using the adaptive notch filter approach. Figure 10b shows the comparison of the separated signal spectrum and the original signal spectrum. To compare the proposed method with the adaptive notch method, we use the signal-to-interference ratio (SIR) to verify. SIR calculation is defined as

(19) $$SIR=10\log_{10}\;\left[\frac{\sum_{n=1}^{N}x{\left(n\right)}^{2}}{\sum_{n=1}^{N}{\left(x\left(n\right)-\hat{x}\left(n\right)\right)}^{2}}\right],$$

where x(n) and $\hat{x}\left(n\right)$ are the original signal component and the corresponding result of CWI suppression at the nth point, respectively. As shown in Table 3, the SIR of the proposed method is 20.0893 dB, while that of the adaptive notch filter method is 20.0954 dB. The adaptive notch filter is suitable for removing narrowband interference. It uses the least mean square (LMS) algorithm to adjust parameters adaptively to simulate interference and then subtract. When the notch frequency happens to be the interference frequency, the interference can be removed while retaining the effective signal as much as possible. Contrastively, after selecting the appropriate sparse expression dictionaries for the Loran-C signal and CWI, the proposed method eliminated the small sparse coefficients through forward and reverse transformation by iteration. Then, the inverse transformation of the corresponding dictionary is performed on the reserved sparse coefficients to obtain effective signals and interference. The interference is simulated by the LMS algorithm in the adaptive notch filter method. Similarly, the proposed method in this paper can simulate the interference with the help of dictionaries through the BCR algorithm. Therefore, when selecting suitable dictionaries, we can achieve the effect of the adaptive notch filter method without knowing the interference frequencies. The SIR result of the proposed method is basically equal to that of the adaptive notch filter method. However, when using the adaptive notch filter method to remove the CWI, we directly use the added CWI frequency as a result of frequency estimation. However, in practice, the adaptive notch filter method is not so good because of the frequency point drift. It should be emphasized that the adaptive notch filter method needs to know the frequencies of CWI, which is not required in our proposed method. Therefore, the proposed method is more effective in suppressing the CWI than the adaptive notch filter method. In this way, we can reduce the damage to the effective signal and the incomplete suppression of CWI caused by the inaccurate estimation of CWI frequencies.

### 3.2. Actual Data Example

**Figure 11:**
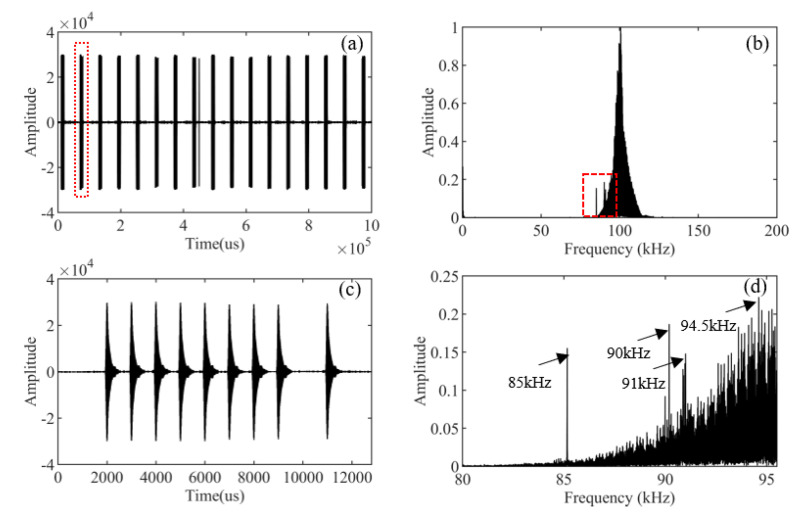
The actual data and spectrum. (**a**) The actual Loran-C signal. (**b**) The actual Loran-C signal spectrum. (**c**) A Loran-C pulse set. (**d**) Details of the spectrum diagram.

In order to further verify the effectiveness of the proposed method, we apply it to the actual data. Figure 11a is the actual Loran-C signal, and its spectrum is shown in Figure 11b. From the magnified detail of the spectrum part in Figure 11d, we can see that the main CWI frequencies are 85 kHz, 90 kHz, 91 kHz, and 94.5 kHz. As shown in Figure 11c, we intercept a pulse set and apply our method to it.

**Figure 12:**
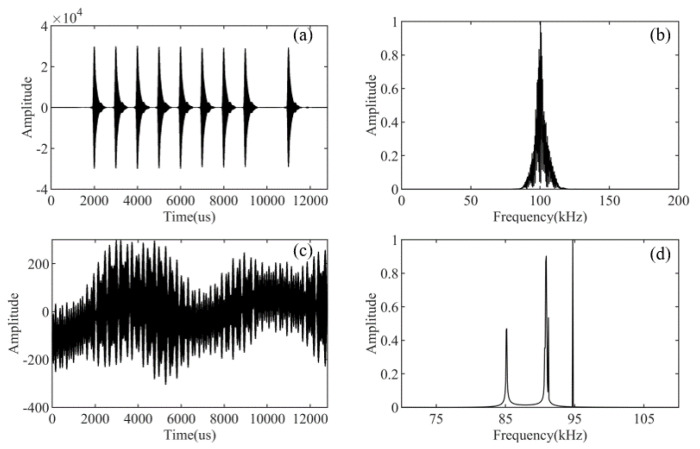
The separation results obtained by the proposed method. (**a**) The separated Loran-C signal. (**b**) The separated Loran-C signal spectrum. (**c**) The separated CWI. (**d**) The separated CWI spectrum.

Figure 12 is the separation result obtained by the proposed method. Figure 12a shows the separated Loran-C signal. Although it is not easy to see the difference in the waveforms, we can still see that the CWI is suppressed effectively in the gap between the pulses. It can also be demonstrated from the Loran-C signal spectrum in Figure 12b. Figure 12c is the separated CWI, and Figure 12d is the spectrum of the separated CWI. It can be seen that the CWI of frequencies 85 kHz, 90 kHz, 91 kHz, and 94.5 kHz are separated from the Loran-C signal. The good separation results show that the sparsity-enabled method of constructing dictionaries is both effective and simple. Therefore, our method can effectively suppress the CWI without knowing the frequencies. In practice, if the frequencies of CWI change in the Loran-C signal propagation environment, the proposed method can also cope with it well.

## 4. Conclusions

In this paper, we proposed a sparse-enabled method to suppress continuous wave interference (CWI) in the Loran-C signal. The main idea is to construct dictionaries suitable for Loran-C signals and CWI according to their morphological characteristics of in the morphological component analysis. We use the tunable Q-factor wavelet transform as the sparse representation dictionary of the Loran-C signal and the discrete cosine transform as the CWI dictionary. Then we use the block-coordinate-relaxation method to separate the two components. Compared to the adaptive notch filter method, the proposed method is very effective, and it does not need previous knowledge of the frequencies of CWI and can suppress the white noise. We demonstrate the effectiveness of the proposed method in both synthetic and measured data. 

## Figures and Tables

**Table 1:** The sparsity estimation using the TQWT with Q=10 and the DCT.

	Loran-C Signal	Continuous Wave Interference
TQWT with Q=10	0.0214	0.0764
DCT	0.0195	0.0156

**Table 2:** Comparison of the third zero-crossing position.

SNR	Loran-C Signal	The Composite Signal	The Processed Signal
20	80.0000 us	80.1731 us	80.0181 us
15	80.0000 us	80.1245 us	80.0173 us
10	80.0000 us	80.5043 us	80.0273 us
5	80.0000 us	79.8822 us	80.0456 us
0	80.0000 us	79.7070 us	80.1341 us

**Table 3:** The SIR comparison of the proposed method and the adaptive notch filter method.

	The Proposed Method	Adaptive Notch Filter Method
SIR	20.0893 dB	20.0954 dB
